# Paedomorphosis in the Ezo salamander (*Hynobius retardatus*) rediscovered after almost 90 years

**DOI:** 10.1186/s40851-021-00183-x

**Published:** 2021-12-08

**Authors:** Hisanori Okamiya, Ryohei Sugime, Chiharu Furusawa, Yoshihiro Inoue, Osamu Kishida

**Affiliations:** 1 Field Science Center for Northern Biosphere, Hokkaido University, Takaoka, Tomakomai Japan; 2 Graduate School of Environmental Science, Hokkaido University, Takaoka, Tomakomai Japan; 3 Graduate School of Environmental Science, Hokkaido University, Sapporo, N10W5 Japan

**Keywords:** Caudata, Hynobiidae, Facultative paedomorphosis, Neoteny, Morphology, Sexual maturity, Spermatozoa, Artificial fertilization, Hokkaido

## Abstract

Although paedomorphosis is widespread across salamander families, only two species have ever been documented to exhibit paedomorphosis in Hynobiidae. One of these two exceptional species is *Hynobius retardatus* in which paedomorphosis was first reported in 1924, in specimens from Lake Kuttara in Hokkaido. This population became extinct after the last observation in 1932; since then, no paedomorphs of this species have been reported anywhere. Here, we report the rediscovery of paedomorphs of this species. Three paedomorph-like male salamanders were collected from a pond in the south Hokkaido in December 2020 and April 2021; in size, these specimens were similar to metamorphosed adults but they still displayed larval features such as external gills and a well-developed caudal fin. An artificial fertilization experiment demonstrated that they were sexually compatible with metamorphosed females, thus, confirming them to be paedomorphs. Future efforts to find additional paedomorphs in this and other populations are required to assess the prevalence of paedomorphosis in *H. retardatus* and to improve understanding of the ecology and evolution of paedomorphisis in Urodela.

**Supplementary Information:**

The online version contains supplementary material available at 10.1186/s40851-021-00183-x.

## Background

Paedomorphosis, defined as the retention of larval features while attaining sexual maturity, has long been of great interest in the fields of developmental biology and evolutionary ecology [1, 2], because it is a representative case of heterochronic development as a mechanism of phenotypic diversification [1, 3]. Paedomorphosis is established by either retardation of somatic development relative to sexual maturation (i.e., neoteny) or acceleration of sexual maturation relative to somatic development (i.e., progenesis) [1]. In urodelan amphibians, paedomorphosis is widespread, having been documented in nine of the ten salamander families [1] and the particular processes of paedomorphosis (i.e., neoteny and/or progenesis) were revealed in some specie s[1, 4–6]. Paedomorphosis is also categorized in the regard to expression modes (i.e., obligate or facultative) [7]. In salamanders, four families exhibit obligate paedomorphosis, in which complete metamorphosis does not occur (i.e., metamorphosis has been evolutionarily lost). In the other five families, including Ambystomatidae, Salamandridae and Hynobiidae, paedomorphosis occurs as a polyphenism (i.e., facultative paedomorphosis) [3, 5, 7, 8].

In the Asian salamander family Hynobiidae, which includes 86 recognized species [9, 10], only two species (*Hynobius retardatus* and *Batrachuperus londongensis*) have ever been documented to exhibit facultative paedomorphosis [9, 11–13]. One of these two exceptional species, *Hynobius retardatus*, which is distributed on Hokkaido Island, Japan, is the subject species of this study. *Hynobius retardatus* typically has a biphasic life cycle, with an aquatic larval stage that metamorphoses to a terrestrial adult stage. However, around 100 years ago paedomorphosis was reported in *H. retardatus* inhabiting Lake Kuttara, a small volcanic lake in Hokkaido [11]; specimens collected from the bottom of the lake were sexually mature but exhibited aquatic larval morphology (e.g., external gills and well-developed caudal fins) [11, 12]. Unfortunately, this paedomorphic population had disappeared after the last observation in 1932 (i.e., exact year of the extinction was unknown), apparently eliminated by introduced salmonid fish, and no paedomorphic individuals have ever been reported from other localities [14–16]. Here, we report the rediscovery of paedomorphic individuals of *H. retardatus* after almost 90 years.

**Fig. 1:**
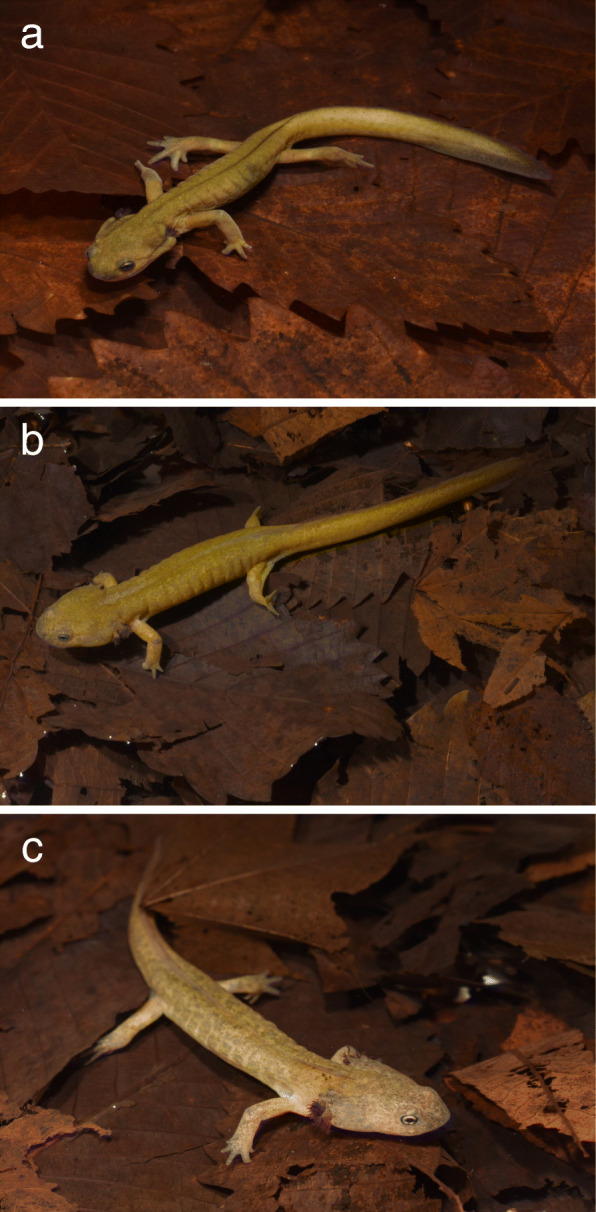
Paedomorph-like individuals of *Hynobius retardatus* in situ. **a** PM-1, **b** PM-2, **c** PM-3

On 1 December 2020 and 5 April 2021, we caught three paedomorph-like individuals (i.e., they were similar in size to metamorphosed adults but possessed larval features) (Fig. 1) in a forest pond in the Iburi region, in south Hokkaido (exact locality withheld for conservation purposes). The pond, elliptical in shape with a mean diameter of 12 m and maximum depth of 1.5 m, was mechanically excavated in 1998. The pond is fed by groundwater and holds water throughout the year. The surrounding habitat is mixed deciduous and coniferous forest, and the closest paved road is approximately 1.5 km away. The pond contains no fish and is used by *H. retardatus* salamanders and *Rana pirica* frogs as a breeding site. In addition, the pond contains an abundance of aquatic invertebrates such as gammaridean amphipods and caddisfly larvae, which are prey for the salamander larvae. The pond is located within 40 km of Lake Kuttara, where the first paedomorphic *H. retardatus* specimens were reported. All three captured paedomorph-like individuals were identified as male by the shape of the cloaca [17]. These individuals were taken to the laboratory at the Tomakomai Experimental Forest of Hokkaido University for morphological assessment and investigation of fertilization. In this paper, we provide evidence that these captured paedomorph-like individuals are in fact paedomorphic *H. retardatus* males.

## Methods

### Morphological characteristics and comparisons

We examined the morphology of all three paedomorph-like males (PM-1, PM-2, and PM-3). For comparison, we collected breeding metamorphosed adults (male, *n* = 10; female, *n* = 10) and overwintering larvae (stage 63 [18]; *n* = 10) from the same pond where the paedomorph-like males were captured. Before the examination, all individuals were fully anesthetized with tricaine methane sulfonic acid (MS-222) solution. We made the following 25 morphological measurements using a digital caliper (i.e., the data were rounded off to the first decimal place): 1) snout-vent length (SVL); 2) head length (HL); 3) head width (HW); 4) maximum head width (MXHW); 5) lower jaw length (LJL); 6) snout length (SL); 7) internarial distance (IND); 8) interorbital distance (IOD); 9) orbit length (OL);10) axilla-groin distance (AGD); 11) trunk length (TRL); 12) tail length (TAL); 13) basal tail width (BTAW); 14) medial tail width (MTAW); 15) basal tail height (BTAH); 16) maximum tail height (MXTAH); 17) medial tail height (MTAH); 18) forelimb length (FLL); 19) hindlimb length (HLL); 20) second finger length (2FL); 21) third finger length (3FL); 22) third toe length (3TL); 23) fifth toe length (5TL); 24) vomerine tooth series width (VTW); and 25) vomerine tooth series length (VTL) (Fig. S1). These morphometric characters have been widely used in salamander taxonomy and for species determination in *Hynobius* (e.g., [19, 20]). The morphometric characters with small absolute values (< 10 mm) were measured under a stereoscopic binocular microscope (Leica MZ95, Leica Microsystems Inc.). To compare the allometric size of morphometric characters among the four life history types (paedomorph-like males, male and female metamorphosed adults, and larvae), the character ratio of each morphometric character to SVL (i.e., as an indicator of the whole body size) was subsequently calculated and expressed as a percentage. We refer to these ratios as relative character values (e.g., RHL, RHW).

To quantify the overall morphological variation of each life history type, we conducted principal component analysis (PCA) using log-transformed relative character values. Then, to analyze the morphological variation among the life history types, we conducted ANOVA on PC1 and PC2 values, followed by Tukey post hoc tests, to reveal the details of the morphological variation.

In addition to the measurable traits (i.e., continuous variables), we visually inspected whether the paedomorph-like males possessed representative morphological features that characterize metamorphosed adults (e.g., eyelids and parotoid glands) or larvae (e.g., external gills and vomerine tooth series). All three paedomorph-like males and several individuals of each of the other life history types were photographed while under anesthesia. All metamorphosed adults and larvae used for the comparison were released at the point of capture after they recovered from the anesthesia.

### Spermatozoa

Two paedomorph-like males (PM-2 and PM-3) and two metamorphosed males (TM-1 and TM-2) were used for spermatozoa observation. The third paedomorph-like male (PM-1) was not used because in our preliminary check for semen emission by gentle pressing on the abdomen, no seminal fluid was discharged. Because no semen was obtained from PM-1, we cannot exclude the possibility that this individual is a large larva rather than a paedomorph. An alternative and reliable method for checking maturation is observation of the gonads by dissection, but we did not adopt this method because lethal operations should not be performed on these rare paedomorph-like individuals. When we gently pressed the abdomens of PM-2 and PM-3, seminal fluid was expelled from the cloacal opening (Fig. S2a). The obtained seminal fluid was then diluted with distilled water and placed on a slide glass for microscopic examination at 250x (SW100, Swift Optical Instruments Inc.). The morphology and motility of the spermatozoa were visually compared between paedomorph-like and metamorphosed males.

### Artificial fertilization

On 16 April 2021, artificial fertilizations were performed to confirm the fertility of the spermatozoa from the paedomorph-like males. We used the two paedomorph-like males with sperm storage (i.e., PM-2 and PM-3), two metamorphosed males (designated TM-1 and TM-2), and six metamorphosed females for the artificial fertilizations. We obtained oocytes from the metamorphosed females by hormonally inducing their ovulation by injecting 400 IU of human chorionic gonadotropin (Aska Pharmaceutical Co., Ltd.) into the abdominal cavity around 24 h before oocyte expulsion. Expulsion of oocytes from the oviduct of each female was facilitated by holding the individuals and gently applying pressure to the abdomen. Scissors were used to divide the pair of egg sacs obtained from each female into three parts. Each part was assigned to one of three treatments: application of paedomorph-like male semen, application of metamorphosed male semen, or control (unfertilized) treatment. Seminal fluid expelled from the males by gently pressing the abdomen was carefully applied to the surface of the eggs with a plastic spatula. This procedure was repeated with three females for each male treatment group (group 1, PM-2 and TM-1; group 2, PM-3 and TM-2). The total number of eggs allocated to each treatment ranged from 24 to 58 (mean ± SD = 40.2 ± 9.9).

The fertilization rate was determined as the proportion of embryos that developed to the gill formation stage (stage 36 [18]), which was attained ten days after fertilization. All embryos that reached the gill formation stage continued to develop and almost all hatched without any abnormality, so the hatching rate was not determined separately. Fertilization rates were not compared statistically because of the small sample sizes.

## Results

### Morphological characteristics and comparisons

**Fig. 2:**
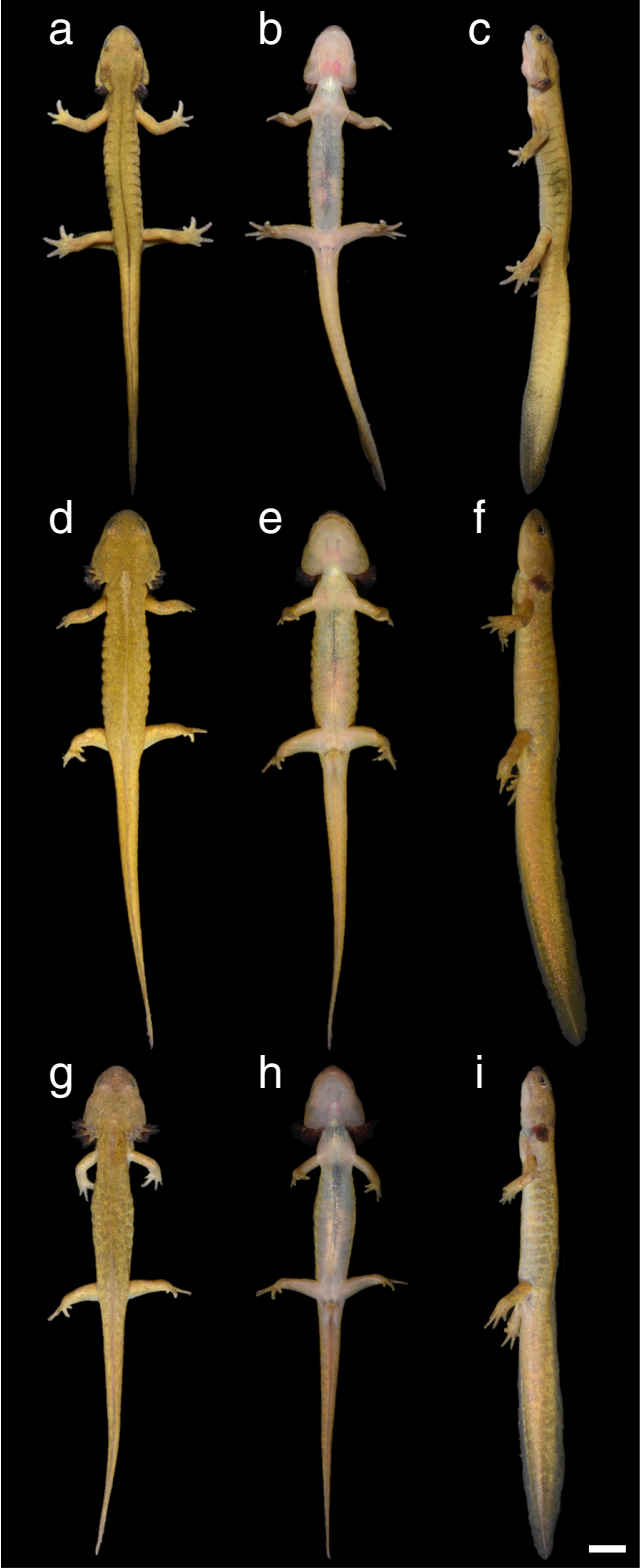
Paedomorph-like individuals of *Hynobius retardatus* in life. Dorsal, ventral, and lateral views of **a–c** PM-1, **d–f** PM-2, and **g–i** PM-3. Scale bar: 10 mm. The Y-shaped cloaca openings of these individuals suggest their maturity [17]

**Fig. 3:**
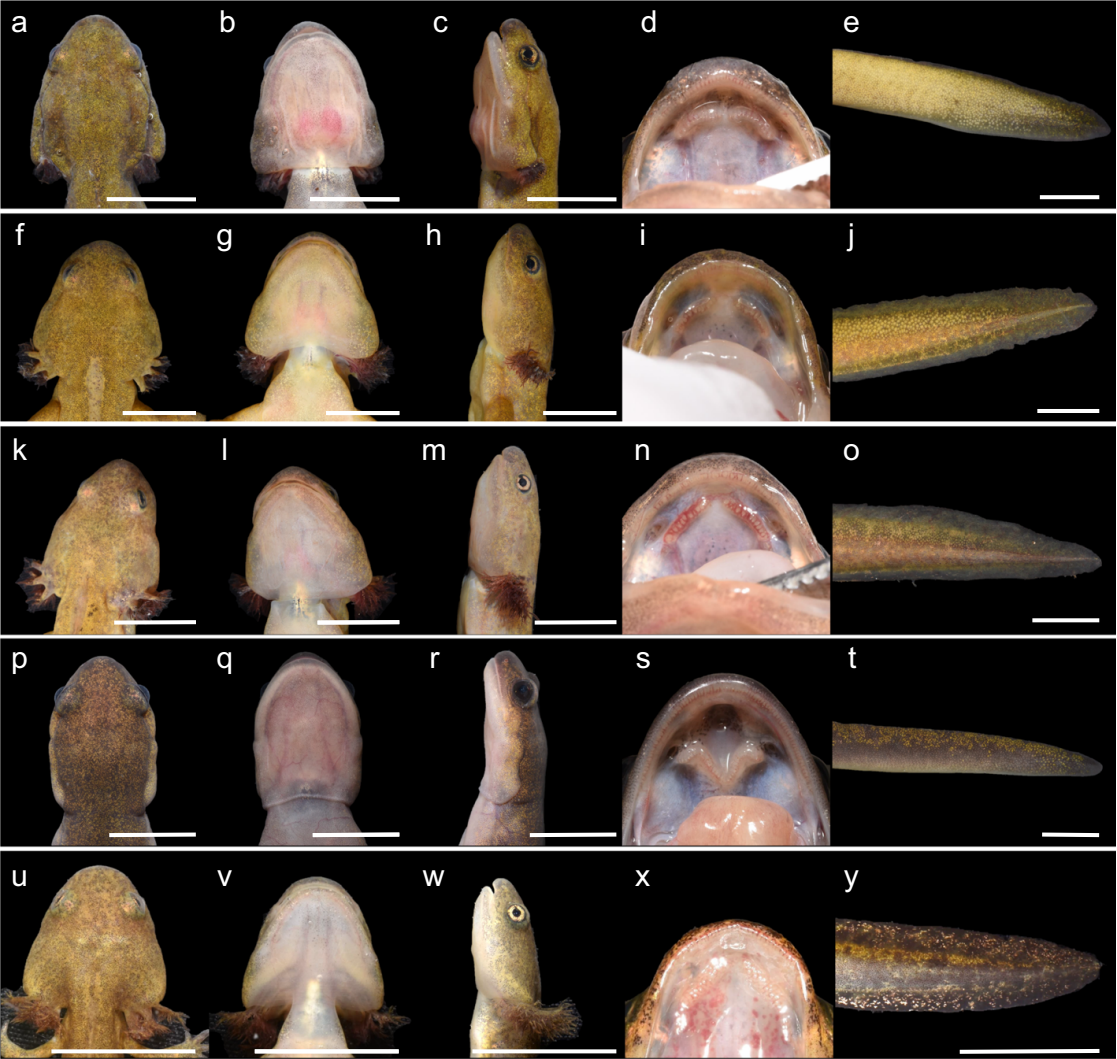
Morphological comparisons of paedomorph-like males, a metamorphosed male, and a larva of *Hynobius retardatus*. Ventral view of head, dorsal view of head, lateral view of head, vomerine tooth series, and lateral view of tail tip of **a–e** PM-1, **f–j** PM-2 **k–o** PM-3 **p–t** a metamorphosed male, and **u–y** a larva. Scale bars: 10 mm

The external morphology of the three paedomorph-like males is shown in Figs. 2 and 3, and the measurements are presented in Table S1. The SVLs of PM-1, PM-2, and PM3 were 58.9, 66.5 and 62.9 mm, respectively (mean ± SD = 62.8 ± 3.8 mm) and their total lengths were 122.2, 143.7 and 130.7 mm, respectively (mean ± SD = 132.2 ± 10.9 mm). In mean size, the paedomorph-like males were 14 and 16% smaller in SVL than the metamorphosed males and females, respectively, and 126% (i.e., 2.26 times) larger than the overwintering larvae.

**Fig. 4:**
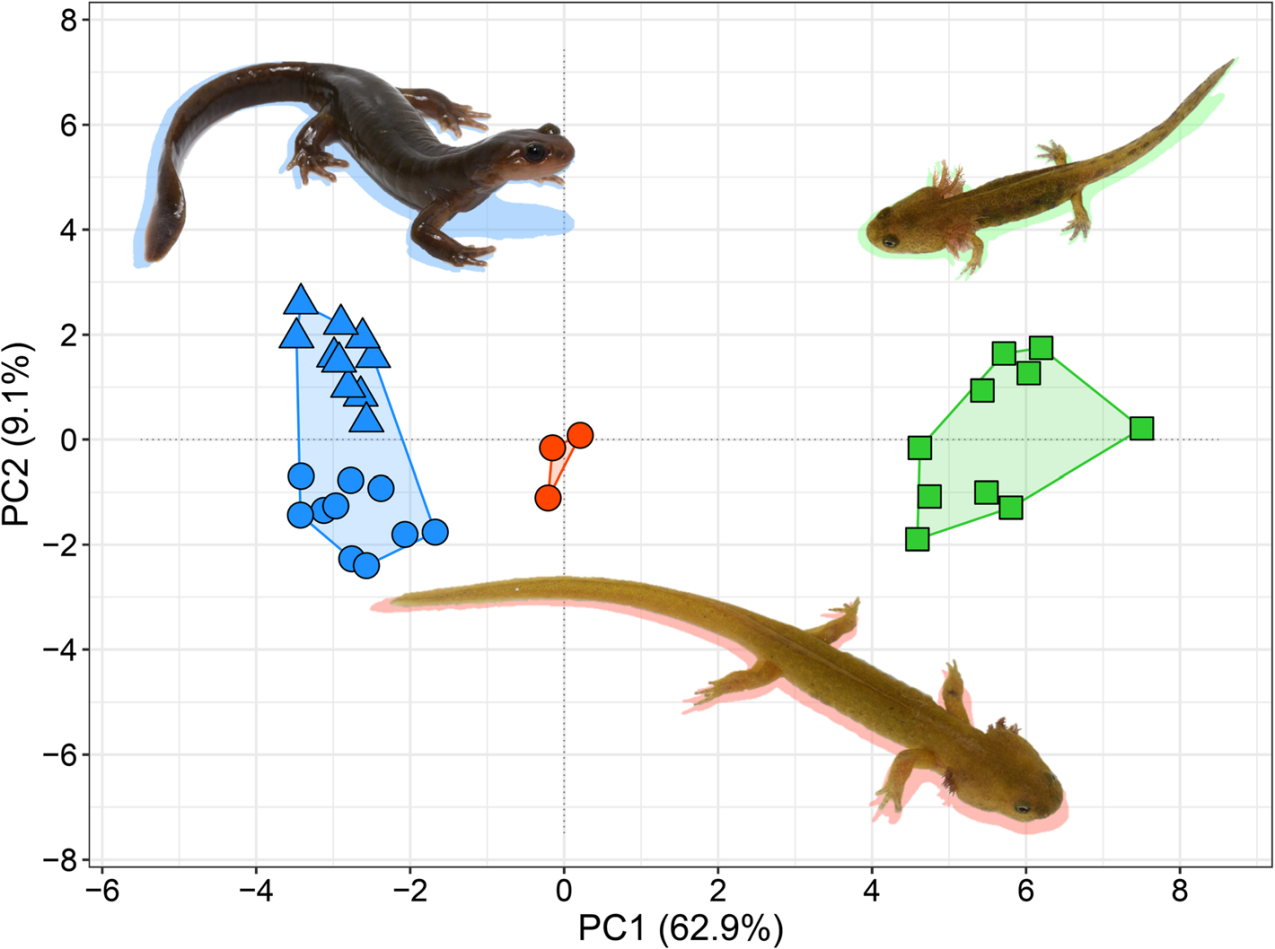
Two-dimensional plot of the principal component analysis results for 25 measured morphological characteristics of the examined paedomorph-like males (orange circles), metamorphosed adult males (blue circles) and females (blue triangles), and larvae (green squares) of *Hynobius retardatus*

In the PCA, PC1 and PC2 explained 62.9 and 9.1%, respectively, of the morphological variation (Fig. 4). The factor loadings of the morphological measurements in the PCA (i.e., eigenvectors) are shown in Table S2. Relative head size and shape (i.e., RHL, RHW, RMXHW, RLJL, RSL, RIND, and RIOD), tail height (i.e., RTAH, RMTAH, and RMXTAH), hindlimb length (i.e., RHLL) and the width and length of the vomerine tooth series (i.e., RVTW and RVTL) had large absolute loadings on PC1 (i.e., eigenvectors of the listed characters were > 0.8), and the relative basal tail width (RBTAW) had a large absolute loading on PC2. One-way ANOVA showed that the variation among the four life history types along both PC1 (F_3,29_ = 414.96, *P* < 0.0001) and PC2 (F_3,29_ = 18.71, *P* < 0.0001) was statistically significant. The Tukey post hoc tests revealed that the paedomorph-like males were statistically distinguishable in PC1 from those of the other life history types. The paedomorph-like males were intermediate in PC1 between those of the metamorphosed adults (both male and female) and the larvae (Fig. S3a). In fact, for most of the morphological characters with strong loadings on PC1 (i.e., measurements characterizing the head and tail shape), the paedomorph-like males exhibited the intermediate value between metamorphosed adults and aquatic larvae (Fig. S4). In contrast, the relative hindlimb length (RHLL) of the paedomorph-like males was similar to that of the larvae and shorter than that of the metamorphosed males. In PC2, the paedomorph-like males were significantly discriminated only from the metamorphosed female (Fig. S3b), but the relative basal tail width (RBTAW), which had the strongest loading on PC2, of the paedomorph-like males was not much different from that of the metamorphosed females (Fig. S4). To summarize, the paedomorph-like males were intermediate between the metamorphosed males and the overwintering larvae with respect to the measured morphometric characters for head and tail shape.

By visual inspection, we observed that the paedomorph-like males possessed certain known larval features of Hynobiid salamanders: namely, external gills and a vomerine tooth series shaped like an inverted V [21]. They possessed external gills, even though their size were relatively small (Figs. 2, 3). PM-2 and -3, like the larvae, had three distinct gill ramus pairs (Figs. 2d, g, 3f, k), but the ramus pairs of the external gills of PM-1 were not distinct (Figs. 2a, 3a). The vomerine tooth series of the paedomorph-like males had an inverted-V or -U shape (Fig. 3d, i, n), resembling the vomerine tooth series of the larvae (Fig. 3x) rather than those of the metamorphosed adults (Fig. 3s). On the other hand, the paedomorph-like males exhibited some morphological features more similar to those of metamorphosed adults rather than to larval features. For example, they showed slightly more developed parotoid glands (Fig. 3a, f, k) than the larvae (Fig. 3u). In particular, PM-1 had evident eyelids and parotoid glands (Fig. 3a, c), similar to those of metamorphosed adults (Fig. 3p, r).

### Spermatozoa

**Fig. 5:**
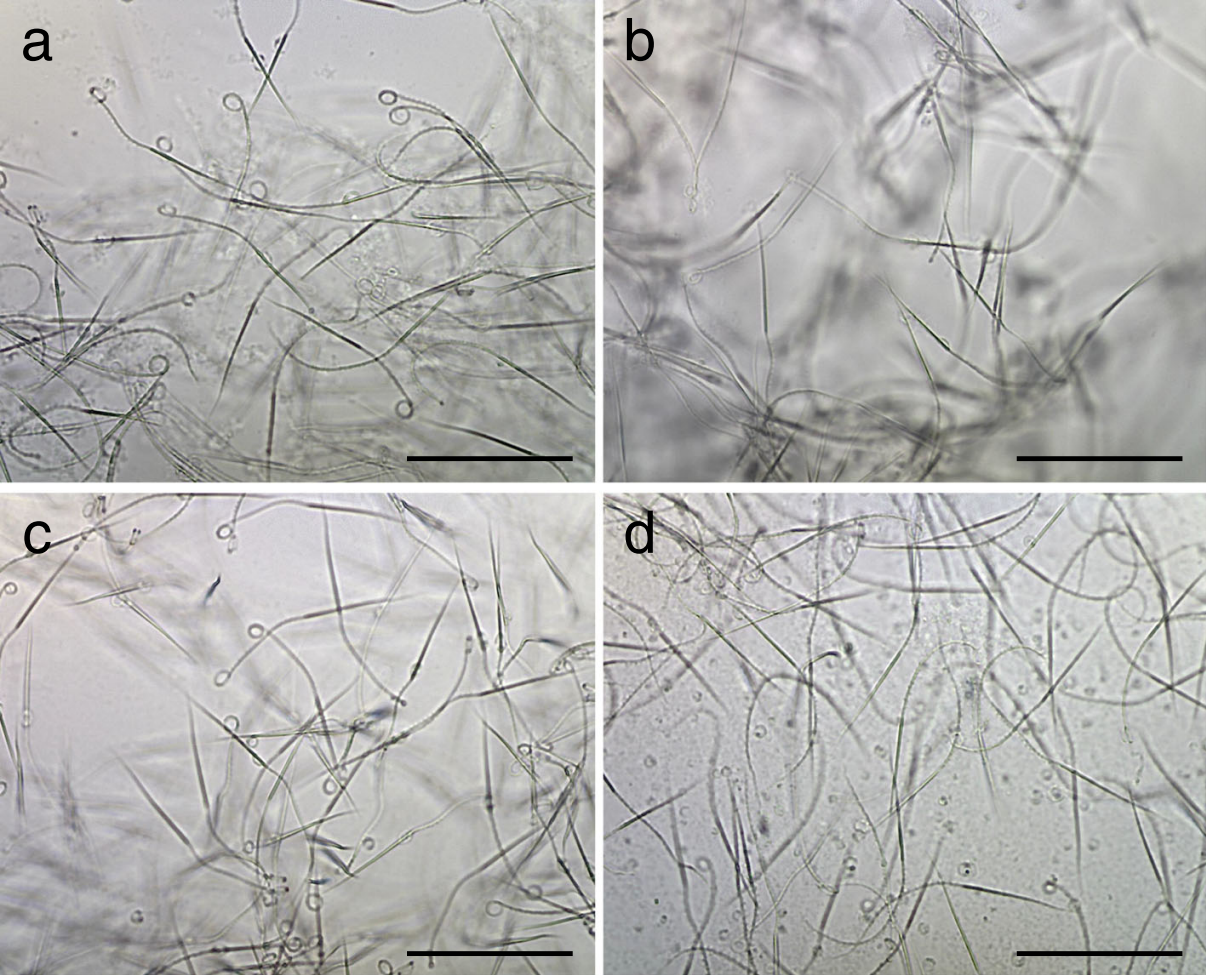
Spermatozoa of paedomorph-like and metamorphosed males of *Hynobius retardatus*. **a, b** Paedomorph-like males (PM-2 and PM-3, respectively). **c, d** Metamorphosed males (TM-1 and TM-2, respectively). Scale bars: 100 μm

Motile spermatozoa were observed in all paedomorph-like males examined, as well as in the metamorphosed males. The size and shape of the spermatozoa were almost identical to those of *Hynobius retardatus* reported previously [22, 23]; approximately 190 μm in total length, with a large lance-shaped head and an axial rod with a wavy flagellum (Fig. 5). No obvious differences in the motility, shape, or size of the spermatozoa were observed between metamorphosed and paedomorph-like males.

### Artificial fertilizations

**Fig. 6:**
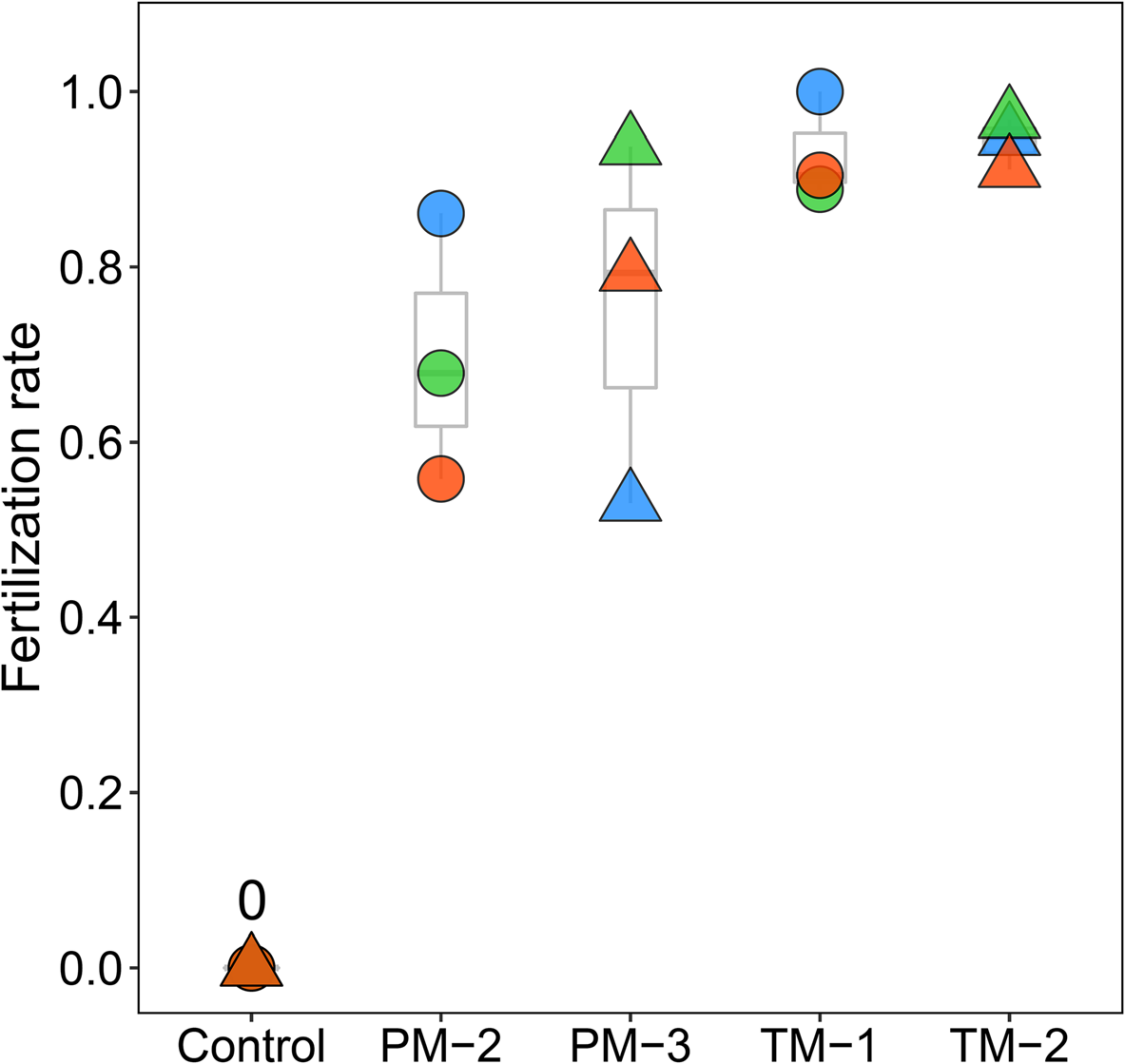
Fertilization rates of paedomorph-like (PM-2 and PM-3) and metamorphosed males (TM-1 and TM-2) obtained by the artificial fertilization experiment. Control indicates unfertilized treatments. Symbols of the same color and shape indicate eggs from the same female parent

Mean (±SD) fertilization rates were 0.69 ± 0.15, 0.75 ± 0.21 0.93 ± 0.06, and 0.94 ± 0.03 for PM-2, PM-3, TM-1, and TM-2, respectively (Fig. 6). Almost all fertilized embryos developed and hatched normally, and no malformations were observed (Fig. S2b, c, d). No eggs developed in any of the control (unfertilized) treatments.

## Discussion

To the best of our knowledge, this is the first report since the paedomorphic population in Lake Kuttara became extinct of paedomorphosis in *Hynobius retardatus*. Because the Lake Kuttara population became extinct soon after its discovery and was mentioned in only a few reports [11, 12], some researchers have been skeptical of the existence of paedomorphosis in *H. retardatus* [14]. In this study, we confirmed the existence of paedomorphosis in *H. retardatus* through detailed morphological comparisons, spermatozoa examination, and artificial fertilizations. The collected paedomorph-like males possessed external gills, a representative larval feature, and their vomerine tooth series had an inverted V or U shape, similar to that of the larvae. In addition, the heads and tails (i.e., caudal fins) of the paedomorph-like males were intermediate in shape between the larvae and the metamorphosed adults, even though the reported morphological differences may be a result of underestimation in the morphological variation of paedomorphs due to their small sample size. These results indicate that the collected paedomorph-like males exhibited larval morphological features. Furthermore, we confirmed that the paedomorph-like males were sexually mature; spermatozoa obtained from two of the paedomorph-like males were motile. Furthermore, our artificial fertilizations demonstrated that the spermatozoa from these paedomorph-like males were able to fertilize the eggs of metamorphosed females and the resulting embryos developed normally. Consequently, we have concluded that these individuals were not large larvae but sexually mature paedomorphic males able to produce spermatozoa with fertilizing capability. As of October 2021, none of them has shown any sign of metamorphosis in the aquarium environment. This suggests that these individuals possess paedomorphic state in multiple years, even though they may metamorphose at later life stage.

We observed one paedomorphic male (PM-2) in the pond on 4 April 2021, during the breeding season; at that time, it was staying, along with some metamorphosed males, at a site in the pond where some metamorphosed females were spawning (Fig. S5). We observed no paedomorphic females in the pond, which suggests that the paedomorphic male was breeding with metamorphosed females. Sexual compatibility between metamorphs and paedomorphs has been reported in other facultative paedomorphic urodelan species [24]. However, it has been suggested that the paedomorphic males may be inferior to metamorphosed males in mating competition due to differences in sexual signaling and reproductive ability [25]. In this study, the fertilization rates of the paedomorphic males were more variable between clutches than those of the metamorphosed males. The paedomorphic males we examined may have expelled less semen than the metamorphosed males and thus may not have been able to fertilize all of the eggs in the each clutch. Low reproductive output of paedomorphs has been reported in other urodelan species [25]. To reduce the burden on the individual salamanders in this study, we did not attempt to control the semen volume but used only the amount of semen that was expelled by gentle pressure on the abdomen. Therefore, we cannot determine whether the observed variation in fertilization rates was due to variation in semen volume or other causes.

Although the body size of the collected paedomorphs in this study (total length = 122.2–143.7 mm) was similar to that reported for the Lake Kuttara population (120–158 mm) [11, 12], we observed some morphological differences. For example, visual assessment of photographs of the Lake Kuttara population [11, 14] revealed that the external gills differed in size between that population and the paedomorphs collected in the present study, with the external gills of the latter being smaller. Our study pond (surface area approximately 100 m^2^ and maximum depth 1.5 m) is much smaller and shallower than Lake Kuttara (i.e., surface area 4,700,000 m^2^ and maximum depth > 140 m). In small and shallow ponds such as our study pond, *H. retardatus* larvae frequently exhibit surfacing behavior to acquire the oxygen from the air (i.e., pulmonary respiration) (Kishida O., personal observation). Thus, the *H. retardatus* paedomorphs in the study pond may rely mostly on pulmonary respiration, which would make large gills for gill respiration unnecessary.

The paedomorphs of *H. retardatus* in Lake Kuttara were described as neoteny [11], but whether paedomorphosis in *H. retardatus* is the result of progenesis (i.e., acceleration of sexual maturation) or neoteny (i.e., reduction of somatic development) has not been verified. Progenetic paedomorphs are usually much smaller than metamorphosed adults, because they reach sexual maturity several years earlier [3, 5]. Furthermore, progenetic paedomorphs are mainly found in temporary ponds. In contrast, neotenic paedomorphs are similar in size to metamorphosed adults and are typically found in permanent waters [5]. The paedomorphs of the Lake Kuttara population as well as those reported here were comparable in size to metamorphosed adults. In addition, the overwintering larvae observed in the study pond were not sexually mature (i.e., their testes and ovaries were undeveloped; Okamiya H., personal observation). Accordingly, we hypothesize that the paedomorphosis in *H. retardatus* is an example of neoteny. In the future, the age structure of the paedomorphs should be assessed by skeletochronology to test this hypothesis.

In the present study, we found paedomorphic males in a pond population dominated by metamorphosed adults. The co-occurrence of paedomorphic and metamorphosed individuals in the same population is typical in facultative paedomorphic species. In such species, larvae either develop into metamorphosed adults through metamorphosis or remain in the aquatic environment to mature as paedomorphic adults, depending on the external and internal conditions that they experience during the larval period [8]. Facultative paedomorphosis was experimentally shown in the paedomorphs collected in Lake Kuttara, which is relatively near our study pond [2, 11]. These findings suggest that the paedomorphs collected in the present study represent facultative paedomorphosis.

## Conclusions

We rediscovered paedomorphs of *H. retardatus* almost 90 years after the last observation. In the past, both male and female paedomorphic salamanders were collected from Lake Kuttara and interbred [11]. In contrast, we found only several individuals of paedomorphic male in our study pond. Thus, efforts to find the paedomorphs in this and other populations are required for further study of paedomorphosis in *H. retardatus*. Since Hynobiidae is a taxonomic group in which paedomorphosis is rare [26], future understanding of the ecological and developmental features of paedomorphosis of *H. retardatus* should provide novel insights into the evolution of paedomorphosis in urodeles.

## Supplementary Information

**Additional file 1.** Paedomorphosis in the Ezo salamander (*Hynobius retardatus*) rediscovered after almost 90 years.

## Data Availability

The datasets used and analyzed in the present study are available from the corresponding author on reasonable request.
