# Supplementary material for: Paedomorphosis in the Ezo salamander (Hynobius retardatus) rediscovered after almost 90 years
Source: Zoological Lett. 2021 Dec 8;7:14. doi: 10.1186/s40851-021-00183-x (PMC8653548; doi:10.1186/s40851-021-00183-x)
Supplement: Supplementary file 1 — Additional file 1. Paedomorphosis in the Ezo salamander (Hynobius retardatus) rediscovered after almost 90 years. [file 40851_2021_183_MOESM1_ESM.docx]

**Additional file 1**

**Paedomorphosis in the Ezo salamander (*Hynobius retardatus*) rediscovered after almost 90 years**

Hisanori Okamiya^1*^, Ryohei Sugime^2^, Chiharu Furusawa^3^, Yoshihiro Inoue^2^ and Osamu Kishida^1^

^1^Field Science Center for Northern Biosphere, Hokkaido University, Takaoka, Tomakomai, Japan

^2^Graduate School of Environmental Science, Hokkaido University, Takaoka, Tomakomai, Japan

^3^Graduate School of Environmental Science, Hokkaido University, N10W5 Sapporo, Japan

*Correspondence: [h.okamiya@gmail.com](mailto:h.okamiya@gmail.com)


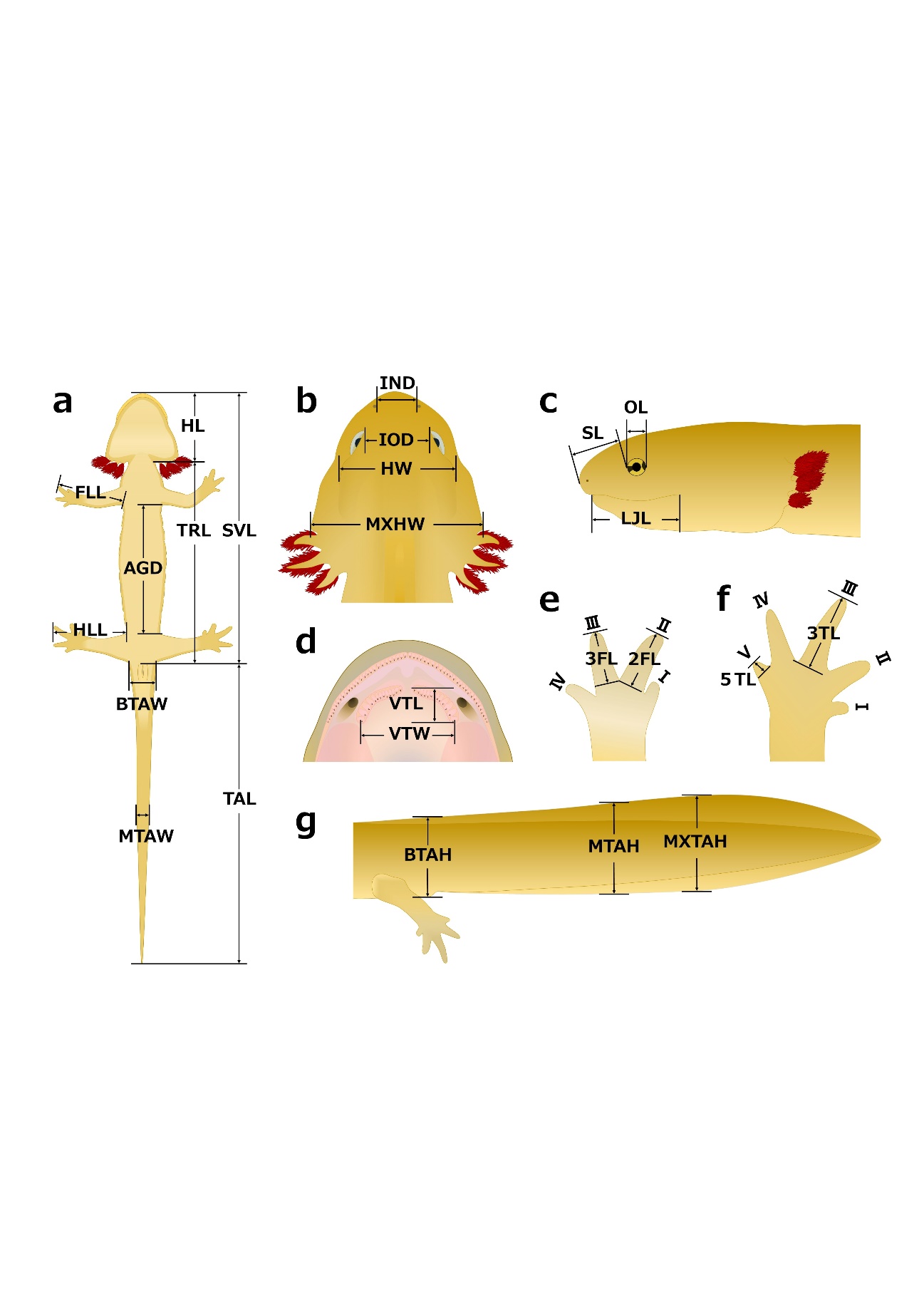


**Figure S1. Morphological characteristics examined in this study.** For abbreviations, refer to Methods. **a** Ventral view of the whole body; **b** dorsal view of the head; **c** lateral view of the head; **d** palatal view of the upper jaw; **e** dorsal view of the left forelimb; **f** dorsal view of the left hindlimb; **g** lateral view of the tail. Roman numerals indicate digit numbers.


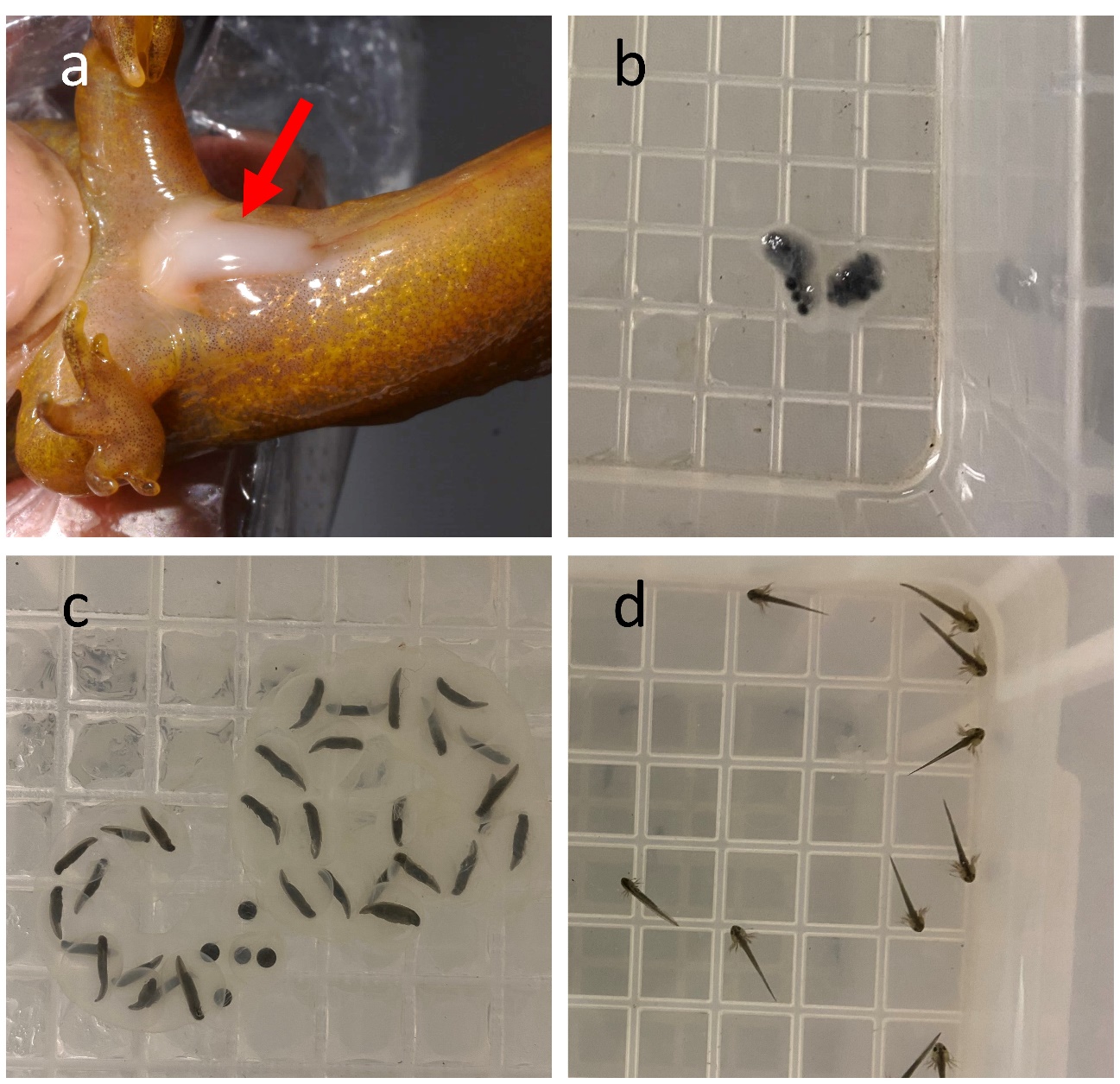
**Figure S2. Artificial fertilization of oocytes from metamorphosed females with spermatozoa from paedomorph-like males. a** Seminal fluid collection from a paedomorph-like male (PM-2). The red arrow indicates expelled semen. **b** Eggs, obtained from a metamorphosed female by hormonal induction, after seminal fluid application. **c** Developing embryos at the gill formation stage. **d** Larvae produced by artificial fertilization of a metamorphosed female with seminal fluid from a paedomorph-like male.


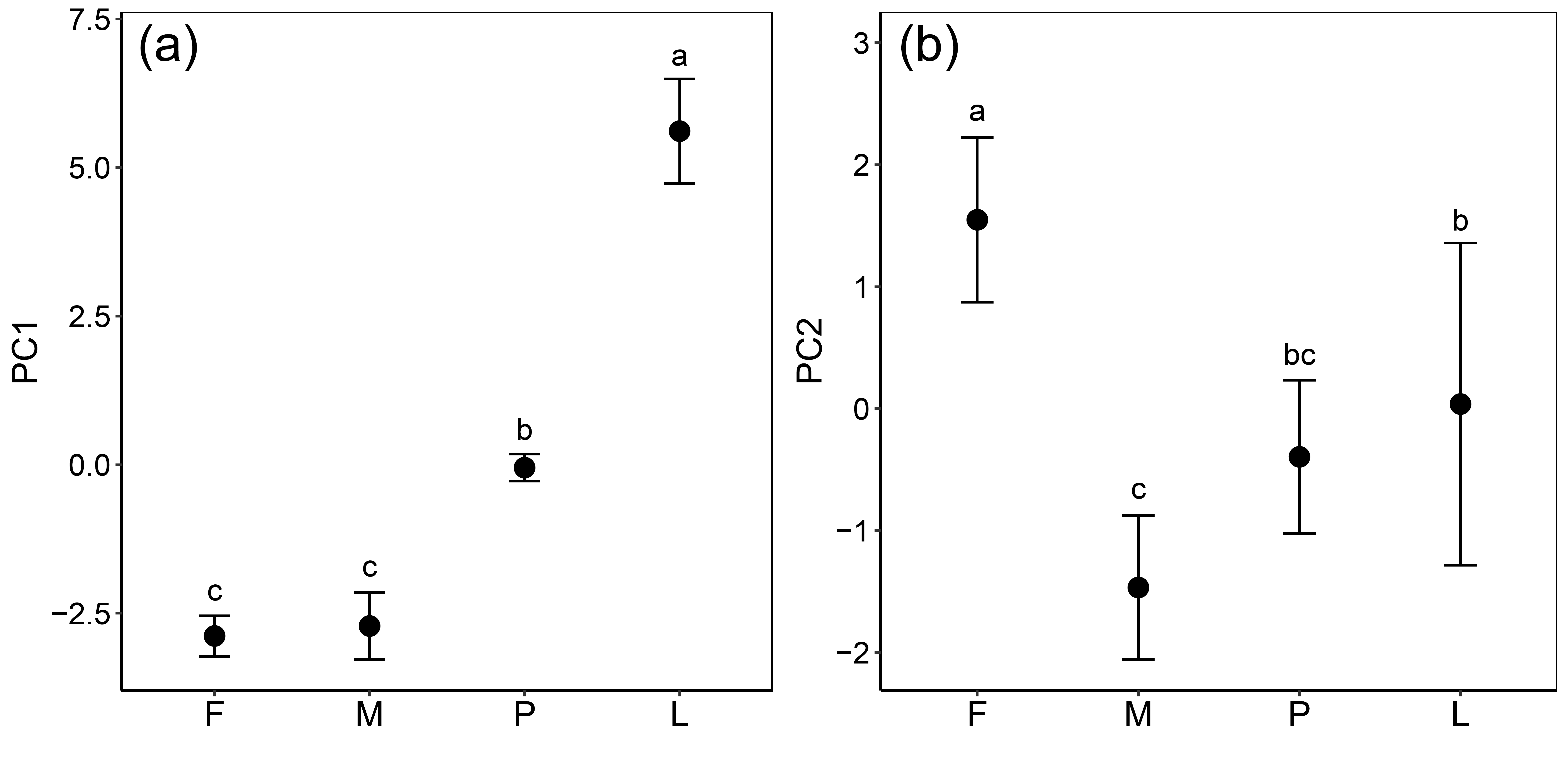
**Figure S3. Principal component analysis (PCA) results for the 25 morphological characters of the four examined life history types of *Hynobius retardatus*.** a PC1, b PC2. F, metamorphosed female; M, metamorphosed male; P, paedomorph-like male; L, larva. Values are means ± SD. Different letters denote significant differences (*P* < 0.05) in the post hoc comparisons.


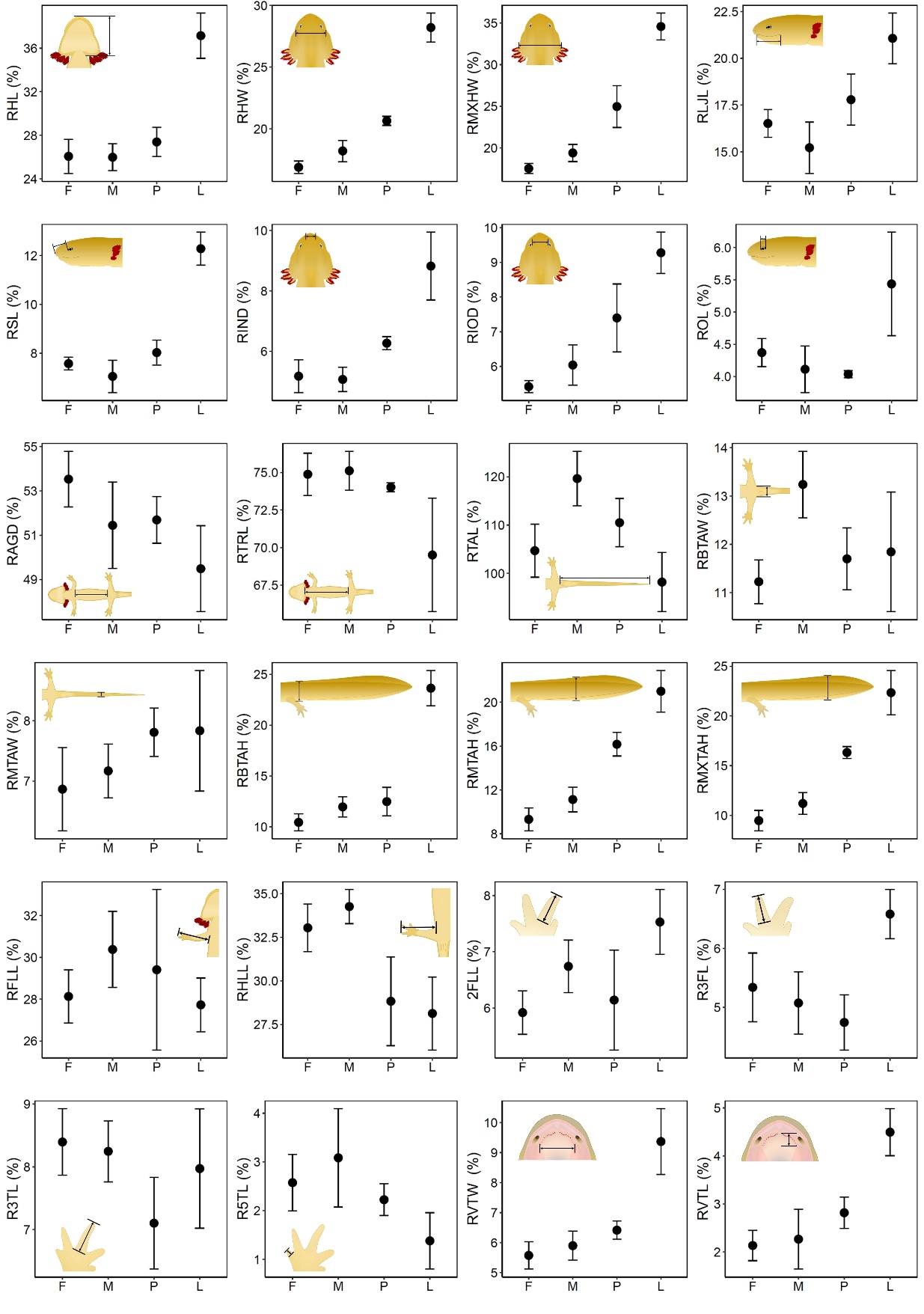


**Figure S4. Metric character ratios (% SVL; mean ± SD) of the four examined life history types of *Hynobius retardatus*.** For character abbreviations, refer to Methods. F, metamorphosed female; M, metamorphosed male; P, paedomorph-like male; L, larva.


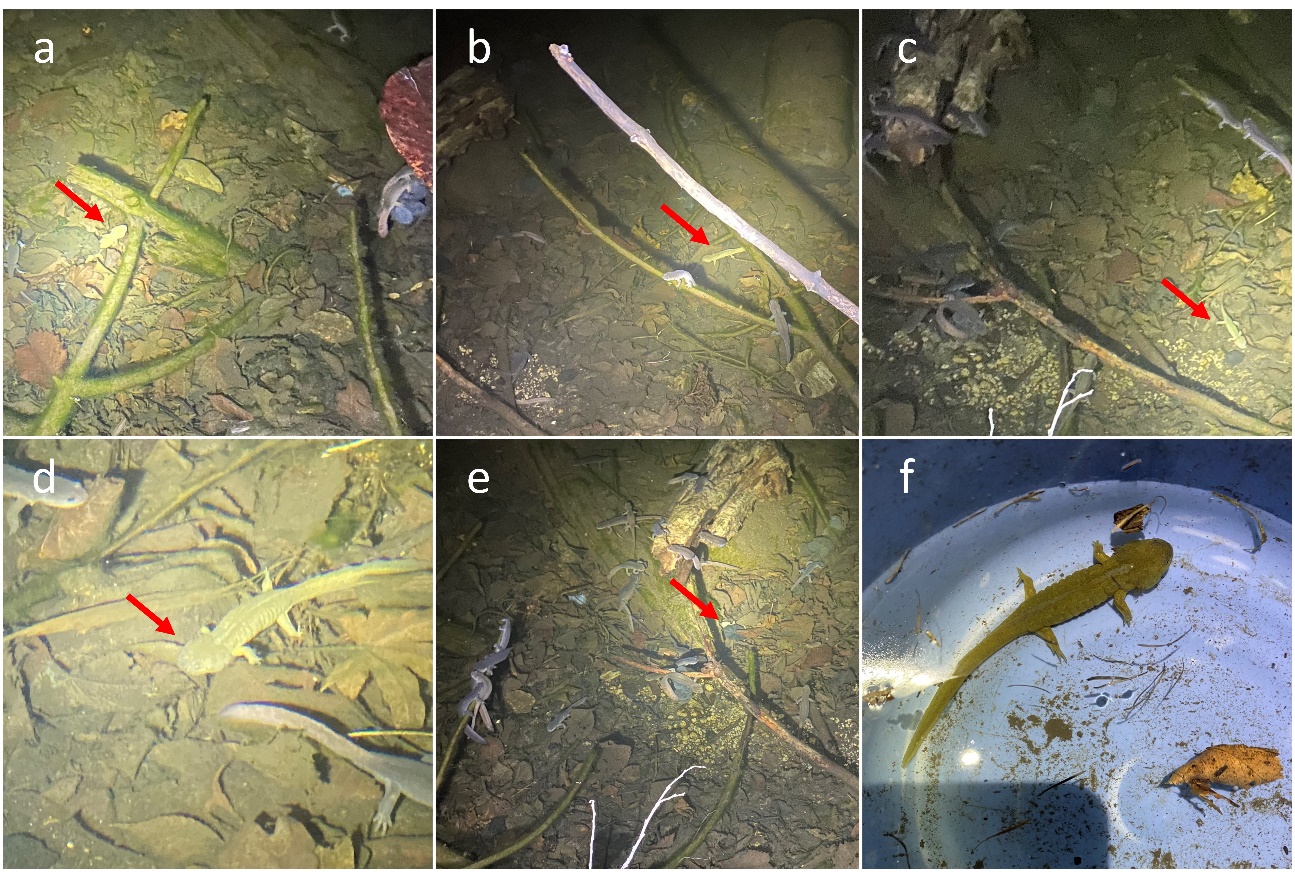


**Figure S5. Natural behavior of the paedomorph-like male.** The natural behavior of PM-2 was observed from 21:59 to 23:18 on 4 April 2021, the day before its capture. **a–e** Photographs of PM-2 at the spawning site in the pond with other breeding males during the observation period. Red arrows indicate PM-2. **f** PM-2 was captured at the spawning site at 20:49 on 5 April 2021. PM-3 was also captured near the spawning site on the same day. PM-1 had been captured in the central part of the pond on 1 December 2020.

**Supplementary Table S1. SVL (mm) and metric character ratios (% SVL) of *Hynobius retardatus*.** For character abbreviations, refer to Methods. N, sample size

| **Category** | Metamorphosed male | | Metamorphosed female | | Larva | | Paedomorph-like male | |
| --- | --- | --- | --- | --- | --- | --- | --- | --- |
| ***N*** | 10 | | 10 | | 10 | | 3 | |
|  | **Mean ± SD** | **Range** | **Mean ± SD** | **Range** | **Mean ± SD** | **Range** | **Mean ± SD** | **Range** |
| **Metric character (mm)** | |  |  |  |  |  |  |  |
| **SVL** | 73.3 ± 2.6 | (69.7–78.2) | 74.6 ± 4.3 | (71.3–85.4) | 27.8 ± 2.2 | (24.3–30.9) | 62.8 ± 3.8 | (58.9–66.5) |
| **Character ratio (% SVL)** | |  |  |  |  |  |  |  |
| **RHL** | 26.0 ± 1.2 | (23.9–27.5) | 26.1 ± 1.6 | (22.5–27.8) | 37.1 ± 2.1 | (33.2–39.5) | 27.4 ± 1.3 | (25.9–28.3) |
| **RHW** | 18.2 ± 0.9 | (16.9–19.4) | 16.9 ± 0.5 | (16.2–17.7) | 28.2 ± 1.2 | (25.9–30.0) | 20.6 ± 0.4 | (20.3–21.1) |
| **RMXHW** | 19.4 ± 1.0 | (17.6–20.7) | 17.5 ± 0.6 | (16.6–18.3) | 34.6 ± 1.6 | (31.7–37.4) | 25 ± 2.5 | (22.2–27.2) |
| **RLJL** | 15.2 ± 1.4 | (12.5–16.9) | 16.5 ± 0.7 | (15.4–17.5) | 21.1 ± 1.4 | (18.4–22.7) | 17.8 ± 1.4 | (16.8–19.4) |
| **RSL** | 7.1 ± 0.7 | (5.6–7.7) | 7.6 ± 0.3 | (7.0–8.0) | 12.3 ± 0.7 | (11.5–13.6) | 8.0 ± 0.5 | (7.5–8.5) |
| **RIND** | 5.1 ± 0.4 | (4.6–5.6) | 5.2 ± 0.5 | (4.4–6.3) | 8.8 ± 1.1 | (6.8–10.2) | 6.3 ± 0.2 | (6.1–6.5) |
| **RIOD** | 6.0 ± 0.6 | (5.1–6.8) | 5.4 ± 0.2 | (5.1–5.7) | 9.3 ± 0.6 | (8.4–10.1) | 7.4 ± 1.0 | (6.3–8.1) |
| **ROL** | 4.1 ± 0.4 | (3.6–4.7) | 4.4 ± 0.2 | (4.0–4.7) | 5.4 ± 0.8 | (4.5–6.4) | 4.0 ± 0.1 | (4.0–4.1) |
| **RAGD** | 51.4 ± 2.0 | (48.3–54.7) | 53.5 ± 1.3 | (52.0–55.9) | 49.5 ± 1.9 | (46.0–52.4) | 51.7 ± 1.1 | (50.7–52.8) |
| **RTRL** | 75.1 ± 1.3 | (73.3–77.0) | 74.9 ± 1.4 | (72.6–77.0) | 69.5 ± 3.8 | (61.9–73.5) | 74.0 ± 0.3 | (73.7–74.2) |
| **RTAL** | 119.6 ± 5.7 | (109.4–127.3) | 104.7 ± 5.5 | (97.5–114.4) | 98.2 ± 6.1 | (84.4–104.2) | 110.5 ± 5.0 | (107.3–116.2) |
| **RBTAW** | 13.2 ± 0.7 | (12.3–14.6) | 11.2 ± 0.5 | (10.8–12.1) | 11.8 ± 1.2 | (10.1–13.4) | 11.7 ± 0.6 | (11.1–12.4) |
| **RMTAW** | 7.2 ± 0.4 | (6.5–7.7) | 6.9 ± 0.7 | (5.3–7.6) | 7.8 ± 1.0 | (6.6–9.7) | 7.8 ± 0.4 | (7.5–8.3) |
| **RBTAH** | 12 ± 1.0 | (11.2–14.4) | 10.4 ± 0.8 | (8.8–11.6) | 23.6 ± 1.7 | (20.5–26.3) | 12.5 ± 1.4 | (11.0–13.8) |
| **RMTAH** | 11.1 ± 1.1 | (9.7–13) | 9.3 ± 1.0 | (7.5–11.1) | 21.0 ± 1.9 | (18.0–23.4) | 16.2 ± 1.1 | (15.0–17.2) |
| **RMXTAH** | 11.2 ± 1.1 | (9.7–13.0) | 9.5 ± 1.0 | (7.5–11.1) | 22.3 ± 2.2 | (19.8–27.2) | 16.3 ± 0.6 | (15.6–16.7) |
| **RFLL** | 30.4 ± 1.8 | (26.7–32.9) | 28.1 ± 1.3 | (25.6–30.4) | 27.7 ± 1.3 | (25.9–29.6) | 29.4 ± 3.8 | (26.6–33.8) |
| **RHLL** | 34.3 ± 1.0 | (33.3–36.2) | 33.0 ± 1.4 | (31.2–35) | 28.1 ± 2.1 | (24.3–31.1) | 28.8 ± 2.5 | (26.3–31.4) |
| **R2FL** | 6.7 ± 0.5 | (5.9–7.4) | 5.9 ± 0.4 | (5.2–6.4) | 7.5 ± 0.6 | (6.5–8.2) | 6.1 ± 0.9 | (5.4–7.1) |
| **R3FL** | 5.1 ± 0.5 | (4.1–6.0) | 5.3 ± 0.6 | (4.3–6.1) | 6.6 ± 0.4 | (5.8–7.2) | 4.7 ± 0.5 | (4.4–5.3) |
| **R3TL** | 8.2 ± 0.5 | (7.6–9.2) | 8.4 ± 0.5 | (7.6–9.5) | 8.0 ± 1.0 | (6.2–9.0) | 7.1 ± 0.7 | (6.4–7.8) |
| **R5TL** | 3.1 ± 1.0 | (1.7–4.7) | 2.6 ± 0.6 | (1.7–3.4) | 1.4 ± 0.6 | (0.4–2.3) | 2.2 ± 0.3 | (1.9–2.6) |
| **RVTW** | 5.9 ± 0.5 | (5.3–6.5) | 5.6 ± 0.5 | (4.8–6.4) | 9.4 ± 1.1 | (7.4–11.1) | 6.4 ± 0.3 | (6.2–6.8) |
| **RVTL** | 2.3 ± 0.6 | (1.6–3.4) | 2.1 ± 0.3 | (1.8–2.7) | 4.5 ± 0.5 | (3.9–5.4) | 2.8 ± 0.3 | (2.6–3.2) |

**Supplementary Table S2. Factor loadings on the principal components (PC 1, 2, and 3) obtained by principal component analysis of the metric character ratios.** For character abbreviations, refer to Methods

| **Character ratio** | **PC1** | **PC2** | **PC3** |
| --- | --- | --- | --- |
| **RHL** | 0.95 | –0.01 | 0.18 |
| **RHW** | 0.98 | –0.10 | 0.03 |
| **RMXHW** | 0.97 | –0.08 | –0.03 |
| **RLJL** | 0.87 | 0.30 | –0.01 |
| **RSL** | 0.95 | 0.11 | 0.11 |
| **RIND** | 0.93 | 0.10 | –0.12 |
| **RIOD** | 0.93 | –0.18 | –0.03 |
| **ROL** | 0.77 | 0.36 | 0.05 |
| **RAGD** | –0.65 | 0.33 | 0.03 |
| **RTRL** | –0.77 | 0.05 | –0.07 |
| **RTAL** | –0.60 | –0.58 | –0.20 |
| **RBTAW** | –0.17 | –0.88 | 0.16 |
| **RMTAW** | 0.43 | –0.52 | –0.16 |
| **RBTAH** | 0.96 | –0.12 | 0.07 |
| **RMTAH** | 0.93 | –0.22 | –0.11 |
| **RMXTAH** | 0.95 | –0.18 | –0.12 |
| **RFLL** | –0.34 | –0.39 | –0.07 |
| **RHLL** | –0.83 | –0.11 | 0.34 |
| **R2FL** | 0.67 | –0.29 | 0.13 |
| **R3FL** | 0.72 | 0.13 | 0.48 |
| **R3TL** | –0.29 | –0.05 | 0.85 |
| **R5TL** | –0.74 | –0.17 | 0.34 |
| **RVTW** | 0.90 | –0.12 | 0.30 |
| **RVTL** | 0.89 | –0.06 | –0.02 |
